# Gastric Cancer Risk Prediction Using an Epidemiological Risk Assessment Model and Polygenic Risk Score

**DOI:** 10.3390/cancers13040876

**Published:** 2021-02-19

**Authors:** Boyoung Park, Sarah Yang, Jeonghee Lee, Il Ju Choi, Young-Il Kim, Jeongseon Kim

**Affiliations:** 1 Department of Cancer Control and Population Health, Graduate School of Cancer Science and Policy, National Cancer Center, Goyang-si 10408, Korea; hayejine@hanmail.net; 2 Department of Preventive Medicine, College of Medicine, Hanyang University, Seoul 04763, Korea; 3 Department of Cancer Biomedical Science, Graduate School of Cancer Science and Policy, National Cancer Center, Goyang-si 10408, Korea; sarahyang@snu.ac.kr (S.Y.); jeonghee@ncc.re.kr (J.L.); 4 Center for Gastric Cancer, National Cancer Center Hospital, National Cancer Center, Goyang-si 10408, Korea; cij1224@ncc.re.kr (I.J.C.); 11996@ncc.re.kr (Y.-I.K.)

**Keywords:** stomach neoplasm, risk assessment, polygenic risk score

## Abstract

**Simple Summary:**

Risk prediction models incorporate various established risk factors to estimate individual risk specifically in cancer. These models additionally include biological or genetic risk factors to assess cancer risk more accurately. The polygenic risk score (PRS) combines the effects of multiple single-nucleotide polymorphisms (SNPs) that are associated with disease; its discrimination ability was assessed both alone and when used in combination with conventional risk prediction models. As few studies have evaluated the combination of genetic variants to identify high risk population of gastric cancer (GC), and we examined the performance of a GC risk assessment model in combination with SNPs as a PRS in consideration of *Helicobacter pylori* (*H. pylori*) infection status. Such a combination improves the identification of a GC-susceptible population among people with *H. pylori* infection.

**Abstract:**

We investigated the performance of a gastric cancer (GC) risk assessment model in combination with single-nucleotide polymorphisms (SNPs) as a polygenic risk score (PRS) in consideration of *Helicobacter pylori* (*H. pylori)* infection status. Six SNPs identified from genome-wide association studies and a marginal association with GC in the study population were included in the PRS. Discrimination of the GC risk assessment model, PRS, and the combination of the two (PRS-GCS) were examined regarding incremental risk and the area under the receiver operating characteristic curve (AUC), with grouping according to *H. pylori* infection status. The GC risk assessment model score showed an association with GC, irrespective of *H. pylori* infection. Conversely, the PRS exhibited an association only for those with *H. pylori* infection. The PRS did not discriminate GC in those without *H. pylori* infection, whereas the GC risk assessment model showed a modest discrimination. Among individuals with *H. pylori* infection, discrimination by the GC risk assessment model and the PRS were comparable, with the PRS-GCS combination resulting in an increase in the AUC of 3%. In addition, the PRS-GCS classified more patients and fewer controls at the highest score quintile in those with *H. pylori* infection. Overall, the PRS-GCS improved the identification of a GC-susceptible population of people with *H. pylori* infection. In those without *H. pylori* infection, the GC risk assessment model was better at identifying the high-risk group.

## 1. Introduction

Risk prediction models incorporate established environmental and behavioral risk factors to estimate individual risk, and numerous models have been developed for cancer. Recently, these models have also included biologic or genetic risk factors to access cancer risk more accurately [1]. Genetic variants have been identified as potential risk factors for cancer, accounting for 15–20% of cases of cancer development [2]. Common sporadic cancers contain a germline genetic component, suggesting that sporadic cancers should be considered polygenic rather than nonhereditary [3]. Among the various types of genetic variants, single-nucleotide polymorphisms (SNPs) prevalent in the general population may be useful in risk stratification to identify at-risk populations. The polygenic risk score (PRS) combines the effects of multiple SNPs associated with disease, and its discrimination ability has been assessed both alone and in combination with conventional risk prediction models [4]. However, these prediction models for cancer mostly target breast cancer, prostate cancer, or colorectal cancer, which are prevalent in Western countries [1,4,5,6,7,8,9,10], whereas models for Asian-prevalence cancers are rare.

Gastric cancer (GC) ranks fifth in cancer incidence and third in cancer mortality worldwide, with over 1.0 million cases and 783,000 deaths in 2018. The incidence and mortality rate of GC vary by region, with highest rates in Eastern Asia regions, including Japan and South Korea, which have the highest incidence and mortality worldwide [11]. According to the Korea Cancer Central Cancer Registry (KCCR) in 2017, the age-adjusted incidence rate of GC was 32.0, 46.4, and 19.6 per 100,000 overall and in men and women, respectively [12]. Environmental and behavioral risk factors for GC have been intensively investigated, and several factors, including *Helicobacter pylori (H. pylori)* infection, obesity, smoking, red meat consumption, salt intake, and alcohol, have been suggested as major causes of GC development [13,14,15,16,17,18]. To date, several large-scale population-based genome-wide association studies (GWASs) have identified common genetic variants associated with susceptibility to GC [19,20,21,22,23,24].

A previous study developed a sex-specific prediction model for GC with 10 known epidemiological risk factors including age, body mass index (BMI), family history of cancer, eating habits, smoking, and physical activity in an East Asian population [25]. Nevertheless, only one study has evaluated a combination of genetic variants to identify populations at high risk of GC [26], and no study has investigated the applicability of a combination of genetic factors with epidemiological risk assessment models. Interestingly, a study conducted in Chinese GC patients reported that the genetic risk score using SNPs related to GC susceptibility might not predict a worse prognosis [27].

Therefore, we evaluated associations between SNPs identified as susceptibility variants for GC in previous GWASs as a PRS on its own or in combination with a GC risk assessment model using traditional risk factors [25] and the risk of GC in an East Asian population according to sex. In addition, associations were further assessed in consideration of *H. pylori* infection status.

## 2. Results

### 2.1. General Characteristics

Table 1 shows the distribution of several risk factors included in the GC risk assessment model for patients and controls. Significant differences were detected for most of the considered risk factors, GC patients, and controls (*p*-value < 0.05), except for BMI and family history of cancer. Regarding *H. pylori* infection status, the rate of positivity was 83.6% in GC patients and 54.7% in controls (*p*-value < 0.001). The odds ratio (OR) of each risk factor included in the GC risk assessment model in univariate analysis is presented in Table S2, as compared with the association from the original study by Eom et al. [25]. The allele frequency distribution and the OR for each SNP included in the PRS are shown in Table S1.

### 2.2. Association between the GC Risk Assessment Model, PRS, and Their Combined Score (PRS-GCS) and GC Risk

Table 2 presents the association between the tertile score of the GC risk assessment model, PRS, their combined score (PRS-GCS), *H. pylori* infection status, and GC based on sex. The ORs of the highest tertile of the GC risk assessment model score were 2.21 (95% confidence interval (CI) = 1.55–3.16) in males and 1.95 (95% CI = 1.24–3.10) in females. The PRS showed a significant association as well (the OR of the highest tertile of the PRS was 1.84 (95% CI = 1.25–2.70) in males and 2.55 (95% CI = 1.58–4.11) in females). Regarding the PRS-GCS, the highest tertile group showed a significantly increased risk for all subjects, males, and females (for the PRS-GCS, the OR of the highest tertile was 2.53 (95% CI = 1.92–3.34) in the total population, 2.60 (95% CI = 1.83–3.71) in males, and 2.67 (95% CI = 1.68–4.31) in females). Associations between *H. pylori* infection and GC were prominent, especially in females, with OR values of 5.48 (95% CI = 3.54 = 8.81) in males and 8.99 (95% CI = 5.13 = 17.08) in females.

Table 3 shows the results of stratified analysis by *H. pylori* infection status. Those with the second highest and highest tertiles of the GC risk assessment model score were more likely to have a risk of GC (OR = 2.61 (95% CI = 0.97–7.75) and OR = 4.15 (95% CI = 1.73–11.56), respectively) if they were negative for *H. pylori* infection, with a modest incremental risk for those positive for *H. pylori* infection (OR = 1.43 (95% CI= 1.04–1.98) and OR = 1.99 (95% CI = 1.45–2.75), respectively). Otherwise, the PRS showed an association only for those with *H. pylori* infection, with an OR of 1.38 (95% CI = 1.03–1.85) for the second highest tertile and 2.19 (95% CI = 1.55–3.10) for the highest tertile. For those without *H. pylori* infection, the PRS did not show an association with GC. When the GC risk assessment model score and PRS were combined, those in the tertile score group showed an increased risk of GC, irrespective of *H. pylori* infection status.

### 2.3. Discrimination Results for the GC Risk Assessment Model, PRS, and Their Combined Score (PRS-GCS)

Discrimination by the GC risk assessment model, PRS, and PRS-GCS presented with area under the receiver operating characteristic curve (AUC) values of 0.580 (95% CI = 0.549–0.612), 0.565 (95% CI = 0.535–0.596), and 0.607 (95% CI = 0.576–0.638), respectively. However, when stratified by *H. pylori* infection status, the PRS did not discriminate GC patients who were negative for *H. pylori* infection, and the GC risk assessment model exhibited modest discrimination for this group (AUC = 0.665 (95% CI = 0.563–0.767)). PRS-GCS discrimination was significant but less than that of the GC risk assessment model in this group. In those with *H. pylori* infection, the AUCs for the GC risk assessment model and PRS were comparable (AUC = 0.574 (95% CI = 0.537–0.611) and AUC = 0.574 (95% CI = 0.539–0.610), respectively). The PRS-GCS showed an increase in discrimination of 3% (AUC = 0.605 (95% CI = 0.569–0.642)) for those with *H. pylori* infection (Table 4).

**Figure 1:**
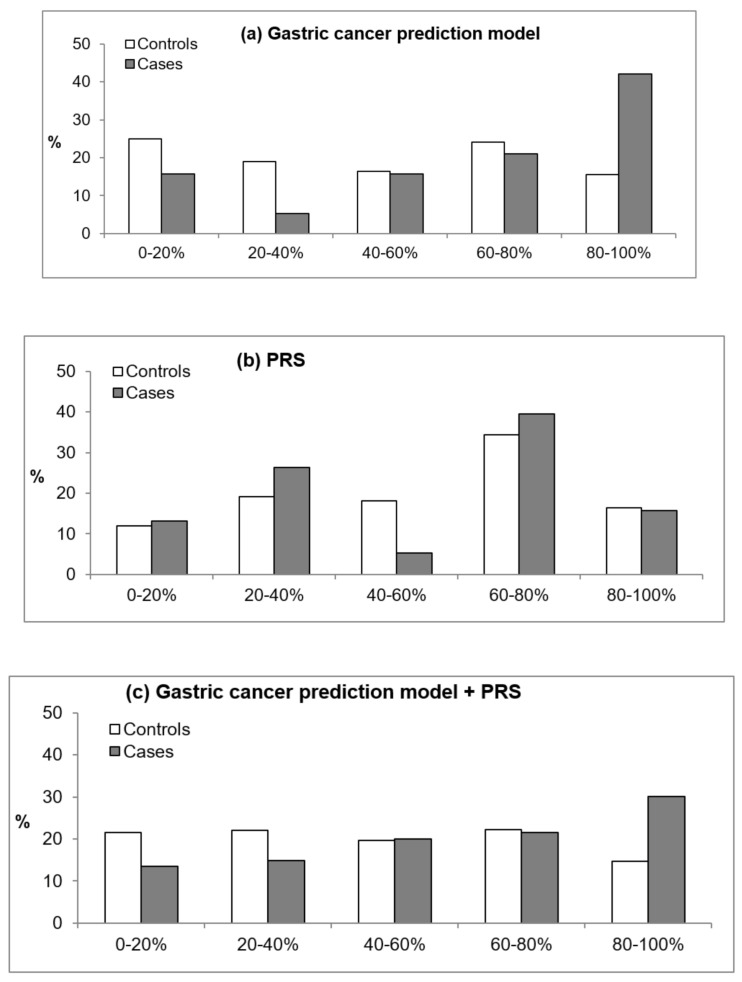
Percentages of patients and controls by quintile estimates according to the estimated score using the gastric cancer prediction model (GCS), unweighted polygenic score (PRS), and PRS-GCS for subjects without *Helicobacter pylori* infection. (**a**) The gastric cancer prediction model, (**b**) the PRS, and (**c**) the gastric cancer prediction model + PRS.

**Figure 2:**
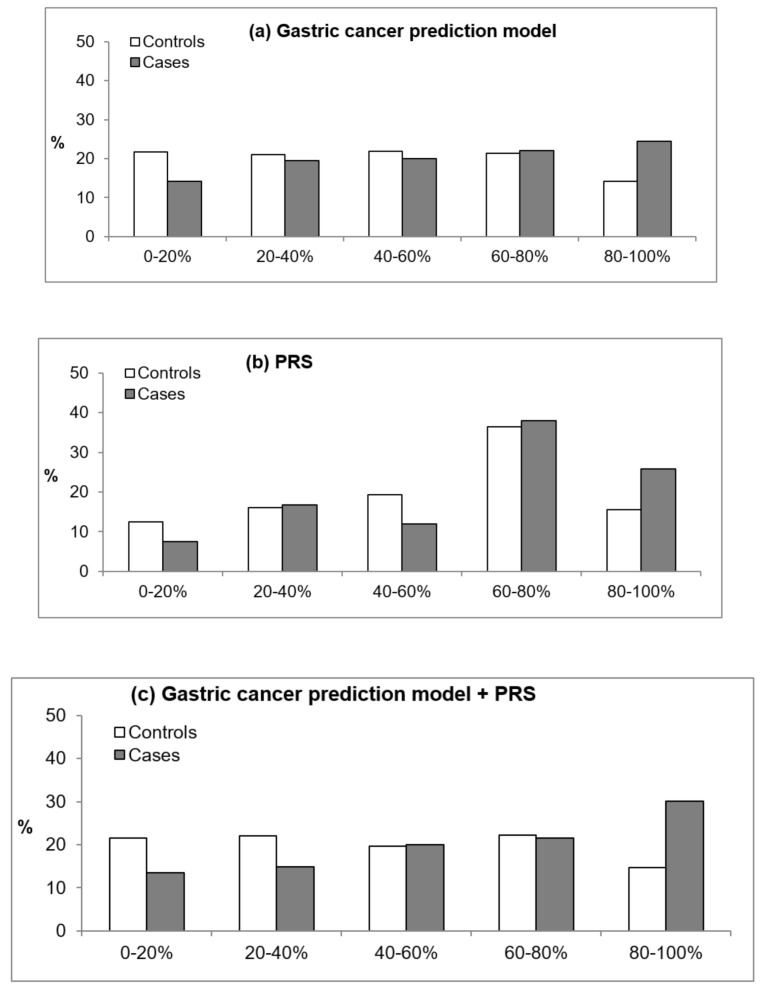
Percentages of patients and controls by quintile estimates according to the estimated score using the gastric cancer prediction model (GCS), unweighted polygenic score (PRS), and PRS-GCS for subjects with *Helicobacter pylori* infection. (**a**) The gastric cancer prediction model, (**b**) the PRS, and (**c**) the gastric cancer prediction model + PRS.

In addition, we evaluated how risk prediction for GC patients and controls differed when the GC risk assessment model, PRS, or GCS-PRS was applied. Figure 1 and Figure 2 present the percentages of patients and controls by quintile estimates according to the GC risk assessment model, PRS, and PRS-GCS based on *H. pylori* infection status. For those without *H. pylori* infection, the GC risk assessment model classified 42% of patients and 15.6% of controls in the highest quintile of the score compared with 15.8% of patients and 16.4% of controls when using the PRS and 30.1% of patients and 14.6% of controls when using the GCS-PRS. These results suggest that patients in the highest quintile were better identified with the GC risk assessment model than when the PRS was applied (Figure 1). In those positive for *H. pylori* infection, the PRS-GCS classified 30% of patients and 14.6% of controls at the highest quintile, which is the greatest difference, compared with 24.5% of patients and 14.2% of controls based on the GC risk assessment model and 25.8% of patients and 15.6% of controls based on the PRS (Figure 2).

## 3. Discussion

To our knowledge, this study is the first to apply the PRS as a genetic risk tool in combination with a conventional epidemiological cancer risk assessment model for GC risk prediction. The PRS, which consists of six SNPs, showed moderate predictive ability for GC in those with *H. pylori* infection; for those without *H. pylori* infection, the PRS did not exhibit significant discrimination. In fact, the conventional epidemiological cancer risk assessment model showed better prediction for this group. When the PRS was combined with the GC risk assessment model, the predictive ability was increased for those positive for *H. pylori* infection but not for those negative for *H. pylori* infection. Compared with the GC risk assessment model or PRS alone, combining the PRS with the conventional risk model classified more cases as high risk (highest quintile) among those positive with *H. pylori* infection.

*H. pylori* infection is the most important cause of GC and is responsible for more than 60% of GC development [28,29]; it has even been suggested that GC occurs only in patients with *H. pylori* infection and not in those without infection [30]. Although risk factors of GC have been studied intensively, the etiology of *H. pylori*-negative GC is less well understood. Some studies have suggested that lifestyle, viral infection, or germline mutations with heritability are associated with GC development [31]. Regardless, one study did not find differences in lifestyle factors, including smoking, drinking, obesity, and family history of GC, as a surrogate for heredity factors between GC patients with and without *H. pylori* infection [32]. In this study, when we assessed environmental and genetic factors as a sum of each effect, the sum of the effect of genetic variants did not show an association with GC in individuals without *H. pylori* infection, even though the sum of the effect of conventional epidemiological risk factors increased GC risk. Our results suggest that in people without *H. pylori* infection, the conventional risk assessment model would well predict GC risk. In contrast, in patients with *H. pylori* infection, the contribution of the conventional risk assessment model and PRS to the prediction of GC development would be similar in terms of increment of OR and AUC. This finding is consistent with the result from a previous study that indicated that some known genetic variants increase GC risk in patients with *H. pylori* infection but show no association in those without *H. pylori* infection [33].

In the GC risk assessment model that we applied in this study, the *H. pylori* infection status was not considered because the information was absent from routine health examination data due to the invasive procedure needed to determine the status, which is the major limitation of the model [25]. In addition, previous studies that applied the polygenic effect on GC development or its prognosis did not consider *H. pylori* infection status [26,27]. This study confirmed that *H. pylori* infection is the most important risk factor for GC, increasing the risk by 7 times, which was comparable to a previous result [34]. These findings suggest that the effect or prediction of genetic factors and environmental factors on GC development would be different according to *H. pylori* infection status.

The PRS based on a large number of markers that did not achieve statistical significance using GWAS data has been applied to several chronic diseases, including obesity, cardiovascular diseases, multiple sclerosis, and psychiatric diseases, with some improvement in prediction [35]. However, in the case of cancer, this approach did not improve risk prediction, and the PRS using established risk SNPs from previous studies showed better performance, suggesting that false-positive markers in the PRS may generate noise with regard to cancer prediction [8]. Thus, among the 12 SNPs genotyped or imputed in this study from 26 GC susceptibility markers in previous GWASs [19,20,21,22,23,24], half with a marginally significant relationship with GC (*p*-value < 0.15) were considered for the PRS. When we compared the PRS using all 12 SNPs irrespective of statistical significance and six SNPs with marginal significance in this study population, the PRS with six SNPs showed better discrimination (data not shown). Replicated SNPs with marginal significance in the study population generate clean signals and represent surrogates that biologically contribute to GC risk in this population [8]. The AUC of the PRS for GC in this study, especially in patients with *H. pylori* infection, was comparable to those for breast, colorectal, and prostate cancers [5,6,7,9,36].

The GC risk assessment model incorporated several modifiable risk factors, such as BMI, eating habits, drinking, smoking, and physical activity, and unmodifiable factors, such as age and family history of cancer, to quantify personal risk. This GC risk assessment model showed good accuracy and predictability, with c-statistics >0.7 [25]. However, the dataset used to develop the GC risk assessment model consisted of a homogeneous population, i.e., Korean government employees, teachers, company employees, and their dependents who underwent medical examination, and the participants were recruited between 1996 and 1997 [25]. In this study population, the AUC of the GC risk assessment model was approximately 0.60 for the total population and 0.67 in those negative for *H. pylori* infection. Reduced performance in individualized risk profiling with a lower AUC has been often shown in other studies of external validation [37,38].

When the PRS was combined with the GC risk assessment model, a modest improvement in discrimination was observed for patients with *H. pylori* infection. This finding has been consistently observed in the application of the risk assessment model for several types of cancer [5,6,10,39]. Although the AUC is a good indicator of discrimination, the result suggests that the predicted risk for an individual with an event is higher than for those without an event, providing limited clinical relevance; additionally, the AUC does not discriminate between individuals with particularly high and low risks [40]. When we classified the estimated risk into quintiles, the PRS-GCS reclassified more GC patients and a smaller proportion of controls into the highest quintile of risk in patients with *H. pylori* infection. Thus, a screening or intervention program that targets those in the highest 20% risk group, as estimated by the PRS-GCS, would capture approximately 30% of cases in the general population, leading to 20–30% improvement compared to the GC risk assessment model alone for patients with *H. pylori* infection, who have a higher risk of developing GC than those without *H. pylori* infection.

There are several limitations of this study. First, among the 26 SNPs selected from previous GWAS, only 12 genotyped or imputed SNPs were used. Thus, some well-established susceptible loci were missed in the analysis, possibly affecting the modest discrimination of the PRS. In addition, the cut-off *p*-value for the inclusion of SNPs (0.15) was arbitrary. Second, the association between epidemiological risk factors and GC risk should be interpreted with caution because, due to the nature of the case-control study, the traditional epidemiological risk factor information measured by questionnaire might have been affected by recall bias, and causal or temporal relationships cannot be guaranteed. Third, when we applied the gastric risk assessment model and PRS according to *H. pylori* infection status, the *H. pylori* type, such as Cag A or Vag A, which are also associated with GC risk [41], was not considered. Fourth, due to the limited sample size, especially for *H. pylori* infection-negative patients, the risk score was divided by tertile or quintile, and more detailed classification could not be conducted. Fifth, despite the preventative effect of *H. pylori* eradication on GC risk [42], we could not incorporate the treatment and eradication of the *H. pylori* among participants with *H. pylori* infection due to a lack of information. However, due to the high eradication rate in the general population in Korea, which was above 65% after 2011 [43], it might be expected that *H. pylori* infection has been eradicated in most of the participants in this study who had been positive. Further studies are needed to incorporate eradication information for risk assessment among those with *H. pylori* infection.

In conclusion, the PRS and GC risk assessment model are independent risk assessment tools for GC, and their combination may improve the identification of a GC-susceptible population, especially among those with *H. pylori* infection, suggesting the importance of both genetic and environmental factors. For those negative for *H. pylori* infection, the GC risk assessment model is applicable only for identifying the high-risk group. More studies to elucidate other genetic variants and clinical applications of them with or without environmental factors should be conducted to identify high-risk groups of GC patients for personalized prevention.

## 4. Materials and Methods

### 4.1. Study Subjects and Genotyping

A total of 450 patients with histologically confirmed GC and 1136 healthy controls who participated in a cancer screening program between April 2011 and December 2014 from the National Cancer Center, Korea, were enrolled in this study. Written informed consent was obtained from all participants. Information on demographic characteristics, lifestyle habits, and dietary intake was collected using a structured questionnaire, and biological samples were obtained. The study protocol was approved by the Institutional Review Board of the National Cancer Center (IRB no. 11-438). Genomic DNA samples of the participants were extracted from peripheral blood leukocytes. The samples were genotyped using an Axiom^®^ Exome 319 chip (Affymetrix Inc., Santa Clara, CA, USA) containing 318,983 polymorphisms. After the standard quality control process and imputation using PLINK v.1.07 [44], SHAPEIT (v2.r837), and IMPUTE2 (2.3.2) with the 1000 Genome Project phase 3 East Asian Ancestry (EAS) sample as a reference panel, 713,348 SNPs were obtained.

Tissue biopsy specimens of the stomach were collected from both the greater and lesser curvatures of the antrum and the body of the stomach through endoscopy examination. The rapid urease test (Pronto Dry; Medical Instruments Corporation, Solothurn, Switzerland) was performed to assess *H. pylori* infection status, which was classified into three categories: negative, positive, and equivocal.

### 4.2. SNP Selection

A total of 26 established susceptible SNPs from previous GWASs were considered [19,20,21,22,23,24]. Among them, data for 12 SNPs with information genotyped using the Axiom^®^ Exome 319 chip were applied (Table S1; rs2294008, rs6656150, rs8280142782, rs760077, rs140081212, rs4460629, rs4072037, rs2274223, rs3765524, rs2285947, rs3781264, and rs11187842).

### 4.3. Risk Factors Used in the Gastric Cancer Risk Assessment Model

We applied a sex-specific GC risk assessment model that was developed for the Korean population. The applied variables included age, BMI, family history of cancer, meal regularity (regular, intermediate, irregular), salt preference (not salty, intermediate, salty), meal preference (vegetable, mixed, meat), weekly meat consumption frequency (≤1 time, 2–3 times, ≥4 times), alcohol consumption, smoking amount, and physical activity (Table S2). The details of the gastric risk assessment model and equation are described in the study by Eom et al. [25].

### 4.4. Statistical Analysis

Of the 12 GC-associated SNPs that were genotyped or imputed for study participants, six associated with GC risk at a *p*-value < 0.15 were included in the PRS (Table S1; rs2294008, rs6656150, rs8280142782, rs760077, rs140081212, and rs4460629). For these six SNPs, the direction of the OR was consistent with a previous GWAS result when we estimated individual SNP association with GC. The ORs of our study population and previous GWAS are compared in Table S1. The PRS of individual i was calculated by the weighted sum of the risk alleles according to the OR from previous GWAS results, as follows:

(1) $$PRS_{i}=\beta_{1}x_{1}+\beta_{2}x_{2}+\ldots \beta_{6}x_{6}$$

where $\beta_{n}$ is the OR for GC of SNP n from previous GWASs [19,20,21,22,23,24], and $x_{n}$ is the number of risk alleles for the SNP n (0, 1, or 2). The OR of each tertile of PRS for GC risk was then estimated.

We compared risk in this study population to that of the previous GC risk assessment model (Table S2). In general, the direction of risk was consistent. The equation from a previously developed model [25] was used to calculate individual risk in this study population. The OR of each tertile of the GC risk assessment model score was estimated. Because *H. pylori* infection status, which is the most important risk factor for GC, was not included in the GC risk assessment model due to a lack of information [25], the association between *H. pylori* infection status and GC was evaluated separately. The analysis was conducted for all of the participants as well as by sex.

To adjust the different ranges of the PRS and risk score from the GC risk assessment model and provide more easily interpretable association results, these two scores were standardized to have a mean of 0 and a variance of 1. Next, the combined PRS and GC risk assessment model score (PRS-GCS) was calculated with each standardized score, and the OR of each tertile was calculated. The AUC was used to compare the discrimination of the GC risk assessment model score, PRS, and PRS-GCS. The analysis was conducted for all of the participants as well as by *H. pylori* infection status (negative and positive). Those with equivocal *H. pylori* infection status were excluded from the subgroup analysis.

## 5. Conclusions

In conclusion, the PRS and GC risk assessment model are independent risk assessment tools for GC, and their combination may improve the identification of a GC-susceptible population, especially in people with *H. pylori* infection, suggesting the importance of both genetic and environmental factors. However, for those negative for *H. pylori* infection, the GC risk assessment model is applicable only for identifying the high-risk group, suggesting that these individuals are more susceptible to environmental factors.

## Figures and Tables

**Table 1:** Basic characteristics of the study population.

Variable	Gastric Cancer Patients	Controls	*p*-Value
	(N = 450)	(N = 1136)	
Sex			<0.001
Male	297 (66.0%)	539 (47.4%)	
Female	153 (34.0%)	597 (52.6%)	
Age, Mean (SD)	55.4 (10.7)	52.1 (8.5)	<0.001
Body mass index (Kg/m^2^)			0.263
<18.5	12 (2.7%)	28 (2.5%)	
18.5–22.9	167 (37.1%)	432 (38.0%)	
23.0–24.9	121 (26.9%)	308 (27.1%)	
≥25	133 (29.6%)	347 (30.5%)	
Missing	17 (3.8%)	21 (1.8%)	
Family history of cancer			0.168
No	227 (50.4%)	561 (49.4%)	
Yes	206 (45.8%)	550 (48.4%)	
Missing	17 (3.8%)	25 (2.2%)	
Meal regularity			0.022
Regular	349 (77.6%)	852 (75.0%)	
Irregular	85 (18.9%)	264 (23.2%)	
Missing	16 (3.6%)	20 (1.8%)	
Salt preference			<0.001
Not salty	46 (10.2%)	233 (20.5%)	
Intermediate	245 (54.4%)	754 (66.4%)	
Salty	143 (31.8%)	128 (11.3%)	
Missing	16 (3.6%)	21 (1.8%)	
Meal preference			<0.001
Vegetables	205 (45.6%)	679 (59.8%)	
Mixed	130 (28.9%)	262 (23.1%)	
Meat	98 (21.8%)	174 (15.3%)	
Missing	17 (3.8%)	21 (1.8%)	
Meat consumption frequency (per week)			0.001
<1 times	73 (16.2%)	206 (18.1%)	
2–3 times	225 (50.0%)	646 (56.9%)	
≥4 times	130 (28.9%)	260 (22.9%)	
Missing	22 (4.9%)	24 (2.1%)	
Alcohol consumption (g/day)			<0.001
0	144 (32.0%)	430 (37.9%)	
1–14.9	137 (30.4%)	444 (39.1%)	
15–24.9	36 (8.0%)	107 (9.4%)	
25 or more	115 (25.6%)	136 (12.0%)	
Missing	18 (4.0%)	19 (1.7%)	
Smoking amount			<0.001
Never	647 (57.0%)	170 (37.8%)	
Ex-smoker	286 (25.2%)	132 (29.3%)	
0.5 pack currently	23 (2.0%)	8 (1.8%)	
0.5–1 pack currently	72 (6.3%)	33 (7.3%)	
1 pack currently	86 (7.6%)	88 (19.6%)	
Missing	22 (1.9%)	19 (4.2%)	
Physical activity			0.009
None	216 (48.0%)	481 (42.3%)	
Low	92 (20.4%)	217 (19.1%)	
Moderate to high	126 (28.0%)	413 (36.4%)	
Missing	16 (3.6%)	25 (2.2%)	
*Helicobacter pylori* infection			
Negative	38 (8.4%)	453 (39.9%)	<0.001
Positive	376 (83.6%)	622 (54.7%)	
Equivocal	36 (8.0%)	61 (5.4%)	

**Table 2:** Association of tertiles of gastric cancer prediction model score, polygenic risk score, *Helicobacter pylori* infection, and gastric cancer.

Prediction Model Score	Total		Male		Female	
	OR	95% CI	OR	95% CI	OR	95% CI
Gastric cancer prediction model score						
1st tertile	1		1		1	
2nd tertile	1.74	(1.31–2.30)	1.46	(1.02–2.10)	2.18	(1.39–3.47)
3rd tertile	2.10	(1.60–2.77)	2.21	(1.55–3.16)	1.95	(1.24–3.10)
Polygenic risk score						
1st tertile	1		1		1	
2nd tertile	1.42	(1.10–1.82)	1.28	(0.93–1.77)	1.79	(1.18–2.72)
3rd tertile	2.03	(1.51–2.72)	1.84	(1.25–2.70)	2.55	(1.58–4.11)
Gastric cancer prediction model score + polygenic risk score						
1st tertile	1		1		1	
2nd tertile	1.48	(1.11–1.97)	1.28	(0.89–1.85)	1.93	(1.20–3.15)
3rd tertile	2.53	(1.92–3.34)	2.60	(1.83–3.71)	2.67	(1.68–4.31)
*Helicobacter pylori* infection ^1^						
Negative	1		1		1	
Positive	7.12	(5.04–10.33)	5.48	(3.54–8.81)	8.99	(5.13–17.08)

^1^ Adjusted for combined gastric cancer prediction model score and polygenic risk score.

**Table 3:** Association of tertiles of the gastric cancer prediction model score and polygenic risk score according to *Helicobacter pylori* infection status.

Prediction Model Score	Total		*Helicobacter pylori* Infection Negative		*Helicobacter pylori* Infection Positive	
	OR	95% CI	OR	95% CI	OR	95% CI
Gastric cancer prediction model score						
1st tertile	1		1		1	
2nd tertile	1.74	(1.31–2.30)	2.61	(0.97–7.75)	1.43	(1.04–1.98)
3rd tertile	2.10	(1.60–2.77)	4.15	(1.73–11.56)	1.99	(1.45–2.75)
Weighted polygenic risk score						
1st tertile	1		1		1	
2nd tertile	1.42	(1.10–1.82)	1.28	(0.61–2.63)	1.38	(1.03–1.85)
3rd tertile	2.03	(1.51–2.72)	1.07	(0.37–1.68)	2.19	(1.55–3.10)
Gastric cancer prediction model score + Weighted polygenic risk score						
1st tertile	1		1		1	
2nd tertile	1.48	(1.11–1.97)	1.20	(0.48–3.01)	1.46	(1.05–2.04)
3rd tertile	2.53	(1.92–3.34)	2.52	(1.15–5.85)	2.43	(1.76–3.38)

**Table 4:** Area under the receiver operating characteristic curve for risk models.

Prediction Model Score	Total		*Helicobacter pylori* Infection Negative		*Helicobacter pylori* Infection Positive	
	AUC	95% CI	AUC	95% CI	AUC	95% CI
Gastric cancer prediction model score	0.580	(0.549–0.612)	0.665	(0.563–0.767)	0.574	(0.537–0.611)
Polygenic risk score	0.565	(0.535–0.596)	0.510	(0.411–0.609)	0.574	(0.539–0.610)
Gastric cancer prediction model score + Polygenic risk score	0.607	(0.576–0.638)	0.605	(0.503–0.708)	0.605	(0.569–0.642)

## Data Availability

The data presented in this study are available on request from the corresponding author.

## Supplementary Materials

The following are available online at https://www.mdpi.com/2072-6694/13/4/876/s1, Table S1: The allele frequency distribution and the odds ratio of each single-nucleotide polymorphism considered for the polygenic risk score in the previous genome-wide association studies and this study, Table S2: Comparison of strength of the association between hazard ratio (HR) in the previous Korean study and odds ratio in this study population.
