# Supplementary material for: Gastric Cancer Risk Prediction Using an Epidemiological Risk Assessment Model and Polygenic Risk Score
Source: Cancers (Basel). 2021 Feb 19;13(4):876. doi: 10.3390/cancers13040876 (PMC7923020; doi:10.3390/cancers13040876)
Supplement: Supplementary file 1 [file cancers-13-00876-s001.pdf]

Supplementary Materials:

Table S1. The allele frequency distribution and the odds ratio of each single nucleotide polymorphism considered for the polygenic risk score in the previous genome-wide association studies and this study

Chr	Position	SNP	Nearest gene	NCC participants							Previous GWAS result			Reference
				Major allele	Minor allele	MAF in cases	MAF in controls	OR	95% CI	P-value	Effect allele	OR	95% CI	
SNPs used for the construction of the polygenic risk score in Asian population with P-value <0.15														
8	143761931	rs2294008	*PSCA*	T	C	0.40	0.52	0.61	0.52-0.72	<0.001	T	1.2	1.15-1.28	Wang et al [9]
1	155123837	rs6676150	-	G	C	0.06	0.10	0.62	0.45-0.84	0.002	C	0.8	0.74-0.86	Helgason et al [7]
1	155485027	rs80142782	*ASH1L*	T	C	0.02	0.04	0.47	0.27-0.81	0.007	C	0.6	0.56-0.69	Wang et al [9]
1	155178782	rs760077	*MTX1, HCN3, MUC1*	T	A	0.09	0.11	0.74	0.56-0.96	0.026	A	0.8	0.73-0.85	Helgason et al [7]
1	155184975	rs140081212	*GBAP1*	G	A	0.09	0.12	0.75	0.57-0.98	0.034	A	0.8	0.73-0.85	Helgason et al [7]
1	155135335	rs4460629	-	C	T	0.10	0.12	0.81	0.62-1.05	0.112	T	0.8	0.67-0.85	Abnet et al [8]
SNPs not used for the construction of the polygenic risk score in Asian population														
1	155162067	rs4072037	*MUC1*	T	C	0.12	0.13	0.86	0.67-1.09	0.200	C	0.7	0.69-0.79	Wang et al [9]
10	96066341	rs2274223	*PLCE1*	A	G	0.27	0.26	1.06	0.89-1.27	0.501	G	1.3	1.19-1.43	Abnet et al [8]
10	96058298	rs3765524	*PLCE1*	C	T	0.26	0.25	1.06	0.89-1.27	0.527	T	1.3	1.20-1.44	Abnet et al [8]
7	21584088	rs2285947	*DNAH11*	G	A	0.32	0.32	1.03	0.87-1.21	0.735	A	1.1	1.08-1.21	Jin et al [4]
10	96070375	rs3781264	*PLCE1*	A	G	0.22	0.21	1.03	0.85-1.25	0.751	C	1.4	1.23-1.50	Abnet et al [8]
10	96052511	rs11187842	*PLCE1*	C	T	0.21	0.21	1.01	0.83-1.22	0.934	T	1.3	1.21-1.49	Abnet et al [8]

MAF; minor allele frequency

Table S2. Comparison of strength of the association between hazard ratio (HR) in the previous Korean study and odds ratio in this study population

	Men		Female	
	HR from the previous study[a] (95% CI)	HR in this study population (95% CI)	HR from the previous study[a] (95% CI)	HR in this study population (95% CI)
BMI				
<18.5	1.09 (1.01-1.18)	2.19 (0.61-8.75)	1.14 (0.99-1.31)	1.07 (0.38-2.56)
18.5-22.9	1	1	1	1
23.0-24.9	0.93 (0.90-0.96)	0.71 (0.49-1.02)	1.02 (0.95-1.09)	1.22 (0.79-1.89)
≥25	0.91 (0.88-0.94)	0.69 (0.49-0.98)	1.00 (0.94-1.06)	1.01 (0.62-1.61)
Family history of cancer				
No	1	1	1	1
Yes	1.32 (1.27-1.37)	0.96 (0.72-1.27)	1.28 (1.02-1.38)	0.89 (0.62-1.28)
Meal regularity				
Regular	1	1	1	1
Intermediate	1.10 (1.06-1.13)		1.01 (0.95-1.07)	
Irregular	1.18 (1.11-1.24)	1.00 (1.00-1.00)	1.06 (0.97-1.15)	1.00 (1.00-1.00)
Salt preference				
Not salty	1	1	1	1
Intermediate	1.08 (1.03-1.12)	1.29 (0.84-2.03)	1.01 (0.94-1.09)	2.29 (1.30-4.31)
Salty	1.24 (1.18-1.30)	4.29 (2.63-7.12)	1.12 (1.02-1.22)	6.91 (3.53-14.19)
Meal preference				
Vegetable	1	1	1	1
Mixed	0.99 (0.96-1.03)	1.42 (1.01-2.00)	0.98 (0.92-1.03)	1.92 (1.26-2.90)
Meat	1.01 (0.96-1.07)	1.70 (1.19-2.46)	1.04 (0.91-1.19)	1.74 (1.03-2.88)
Meat consumption frequency (per week)				
≤ 1 time	1	1	1	1
2-3 times	0.98 (0.95-1.01)	1.11 (0.72-1.74)	0.98 (0.93-1.04)	0.74 (0.48-1.17)

≥ 4 times	1.01 (0.96-1.07)	1.51 (0.95-2.44)	0.97 (0.88-1.08)	0.96 (0.56-1.64)
Alcohol consumption (g/day)				
'0	1	1	1	1
1-14.9	1.06 (1.03-1.11)	0.85 (0.57-1.28)	0.99 (0.91-1.08)	0.81 (0.55-1.19)
15-24.9	1.16 (1.11-1.21)	0.74 (0.44-1.23)	1.24 (1.06-1.45)	1.01 (0.44-1.12)
25 or more	1.33 (1.28-1.38)	1.80 (1.21-2.69)		
Smoking amount				
Never	1	1	1	1
Ex-smoker	1.20 (1.14-1.25)	1.28 (0.84-1.98)	1.27 (1.15-1.39)	1.28 (0.66-2.35)
0.5 pack currently	1.26 (1.20-1.33)	1.45 (0.55-3.61)		
0.5-1.pack currently	1.44 (1.39-1.50)	1.31 (0.73-2.33)		
1 pack currently	1.58 (1.51-1.66)	2.85 (1.78-4.62)		
Physical activity				
None	1	1	1	1
Low	1.10 (0.96-1.04)	0.78 (0.52-1.15)	1.00 (0.94-1.06)	0.64 (0.44-0.92)
Moderate to high	0.95 (0.92-0.98)	0.60 (0.43-0.83)		

[a] Eom BW, Joo J, Kim S, Shin A, Yang HR, Park J, et al. Prediction Model for Gastric Cancer Incidence in Korean Population. PLoS One. 2015;10(7):e0132613
